# New progress and clinical research translation of immune checkpoint inhibitors in systemic therapy of gallbladder cancer

**DOI:** 10.3389/fimmu.2026.1862775

**Published:** 2026-08-06

**Authors:** Fuhong Li, Yuxiao Wang, Zhiren Xu, Meng Zhu, Kun Su, Xiawei Yang, Tao Wu

**Affiliations:** Department of Hepatobiliary Surgery, The Second Affiliated Hospital of Kunming Medical University, Kunming, Yunnan, China

**Keywords:** biliary tract cancer, clinical decision-making, gallbladder cancer, immune checkpoint inhibitors, PD-1, PD-L1

## Abstract

Gallbladder cancer (GBC) is a highly malignant tumor of the biliary tract system.Although it is less common than other malignant tumors of the digestive system, its incidence and mortality rates have been on a continuous rise. With insidious early symptoms, the majority of patients are diagnosed at an advanced stage when the disease is detected. Surgical resection remains the radical treatment for gallbladder cancer yet many patients lose the opportunity for surgical intervention when seeking medical attention due to the onset of symptoms.In addition, given the high invasiveness and high recurrence rate of gallbladder cancer, systemic therapy beyond surgical treatment is particularly crucial. In recent years, precision medicine centered on immunotherapy and targeted therapy has exhibited certain therapeutic effects and improved the quality of life in patients with advanced cancer. Immunotherapy is a therapeutic modality that prevents immune evasion of tumor cells, primarily by means of immune checkpoint inhibitors such as CTLA-4 and PD- 1/PD-L1 inhibitors, among which PD-1/PD-L1 inhibitors are widely used in the immunotherapy of gallbladder cancer.Following the success of the global Phase III TOPAZ- 1 and KEYNOTE-966 trials, durvalumab in combination with gemcitabine and cisplatin, as well as pembrolizumab combined with gemcitabine and cisplatin, have been approved in multiple countries and recommended by authoritative guidelines as the preferred first-line systemic therapeutic regimens for advanced biliary tract cancers. A number of clinical trials conducted in recent years have initiated subgroup analyses of biliary tract cancer together with detections of biomarkers and the tumor microenvironment, providing guidance for immunotherapy of gallbladder cancer. This article elaborates on the mechanisms of immune checkpoint inhibitors, the unique tumor microenvironment and biomarkers specific to gallbladder cancer, summarizes clinical trials concerning gallbladder cancer and those incorporating gallbladder cancer subgroup analyses, interprets authoritative clinical guidelines and relevant policies, and presents the strategies and procedures adopted by our team for administering immunotherapy to patients with gallbladder cancer in routine clinical practice.

## 1 Introduction

**Figure 1:**
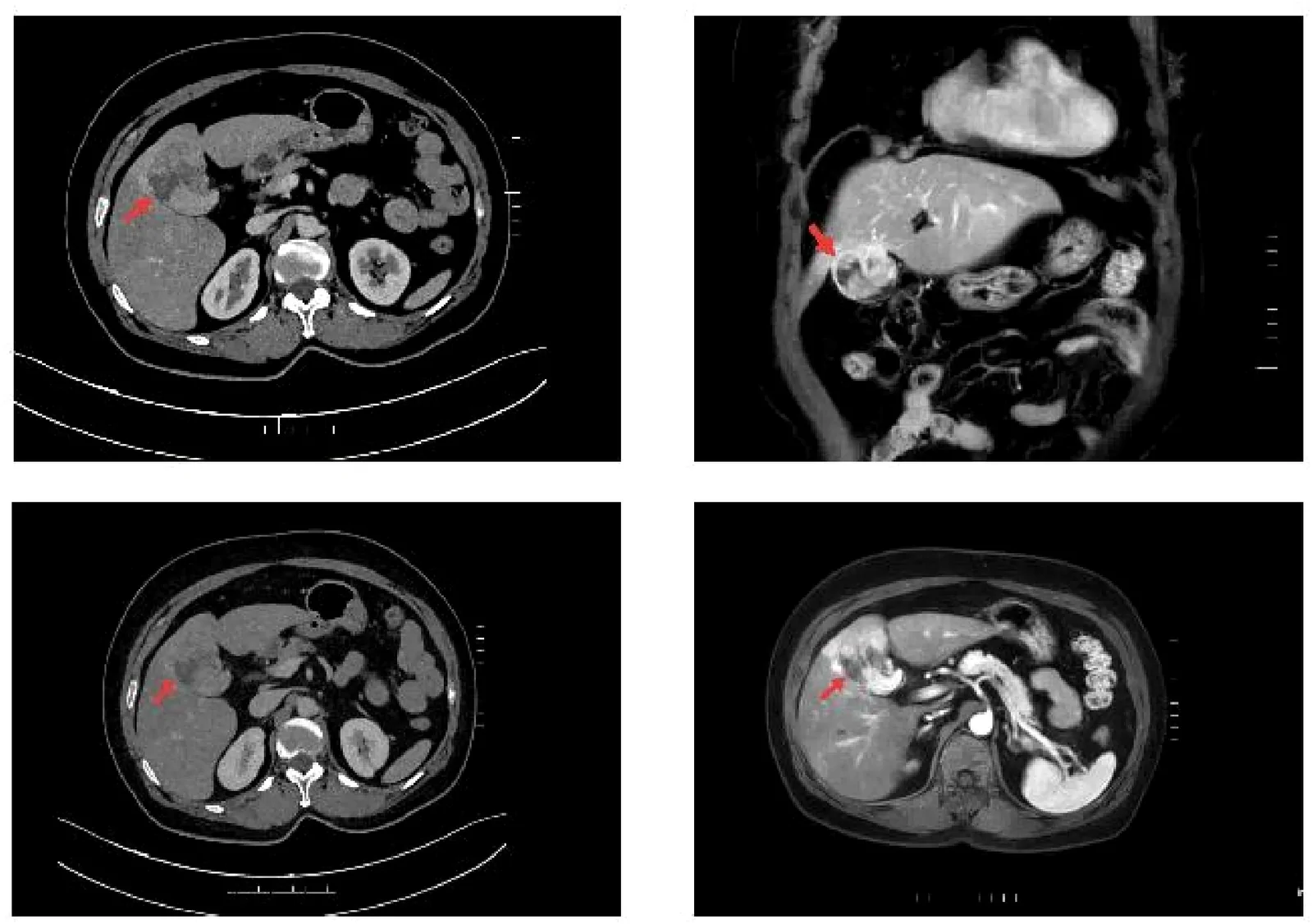
Imaging manifestations of gallbladder carcinoma. The two images on the left are contrast-enhanced CT of the whole abdomen, and the two on the right are contrast-enhanced MRI of the upper abdomen. The regions indicated by red arrows in the images represent the gallbladder carcinoma lesions. Photograph by author.

Gallbladder cancer is a malignant tumor of the biliary tract system originating from the epithelial tissue of the gallbladder mucosal layer, with adenocarcinoma being the most common histological type (1, 2). According to the global cancer data updated by GLOBOCAN 2022, the age-standardized incidence rate of gallbladder cancer is 1.2 per 100, 000 population, and the age-standardized mortality rate is 0.83 per 100, 000 population, both lower than those of several other major malignant tumors of the digestive system (3). However, it is predicted that its incidence and mortality rates will increase significantly in the future (4) gallbladder cancer is characterized by strong concealment, high invasiveness and extremely high malignancy, with a 5-year survival rate of less than 5% for advanced cases (5, 6), while the 5-year survival rate of pancreatic cancer, known as the “king of cancers”, is approximately 3%;15% (7), highlighting the difficulty in the prevention and treatment of gallbladder cancer. Gallbladder cancer often has no definite clinical symptoms in the early stage; most early gallbladder cancer cases are accidentally detected by pathological examination of the gallbladder after cholecystectomy for benign gallbladder diseases, especially gallbladder stones, with the incidence of incidental gallbladder cancer being only 0. 14%;1.6% (8). Therefore, gallbladder cancer is mostly diagnosed at intermediate to advanced stages, and may present with symptoms including right upper abdominal pain, nausea and vomiting, fever, and weight loss, and may also have gallbladder enlargement andjaundice when the bile duct is invaded (9). As shown in **Figure 1**, the imaging findings of gallbladder cancer on contrast-enhanced CT and contrast-enhanced MRI are demonstrated.

At present, surgical resection remains the curative treatment for gallbladder cancer:simple cholecystectomy is feasible for Tis and T1a stages; cholecystectomy plus wedge resection of the liver is mostly performed for T1b stage; cholecystectomy plus resection of liver segments IVb and V is mostly performed for T2a, T2b and T3 stages;palliative surgery may be considered for T4 stage, or cases with multiple regional lymph node metastases or distant organ metastases, for which radical surgery is mostly not an option (10–12). Adjuvant therapy can be administered to patients with unresectable gallbladder cancer, and some patients may thus obtain the opportunity for surgery.The gemcitabine plus cisplatin regimen has become the first-line chemotherapy regimen for advanced gallbladder cancer following the publication of the ABC-02 trial (13–15). Radiotherapy is relatively rare (16) and is mostly used in combination with other treatment regimens to enhance therapeutic efficacy (17, 18). In recent years, targeted therapy against molecular targets such as HER2 amplification, NTRK fusion, EGFR overexpression and BRAF mutation (19–21), as well as immunotherapy targeting immune checkpoints including PD- 1/PD-L1, CTLA4/B7, TIGIT and LAG-3, has achieved progress, providing more treatment options for gallbladder cancer.

## 2 Basis of immunotherapy

### 2.1 Molecular mechanisms underlying the regulation of T cell activation by immune checkpoints

**Figure 2:**
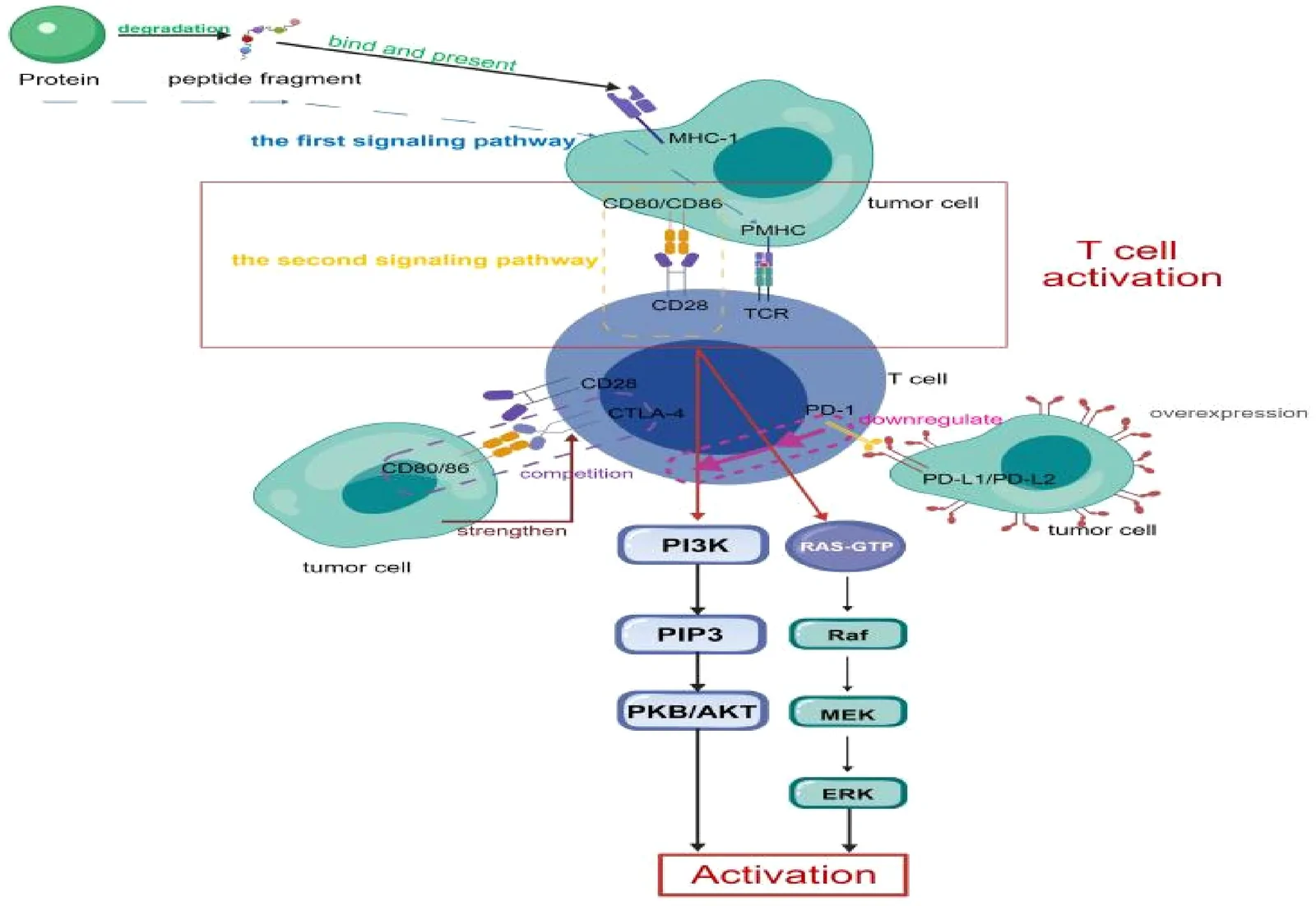
T cell activation and immune checkpoint pathways. T cells are activated through a dual-signal mechanism.The first signal is initiated when peptides generated by protein degradation bind to MHC molecules on tumor cells or antigen-presenting cells (APCs), forming pMHC complexes that subsequently interact with the T-cell receptor (TCR). The second signal is mediated by the engagement of CD80 (B7-1)/CD86 (B7-2) on tumor cells or APCs with CD28 on T cells.CTLA-4/B7 and PD-1/PD-L1 are the two predominant immune checkpoint pathways. CTLA-4 on activated T cells competes with CD28 for CD80/CD86 ligation to deliver co-inhibitory signals, which can be exploited by tumor cells to facilitate immune evasion. Engagement of PD-1 on activated T cells with PD-L1/PD-L2 suppresses the activity of the PI3K/AKT and Ras/MEK/ERK signaling cascades, thereby attenuating effector T-cell activation. Tumor cells achieve immune escape via PD-L1 overexpression. Created with BioGDP.com.

Research on cancer immunotherapy mainly focuses on immune checkpoint inhibitors(ICIs) blocking the activation of immune checkpoints by tumor cells, thereby restoring the anti-tumor effect of the body’s immune system. Immune checkpoints are a large number of inhibitory pathways in the immune system, through which tumors can achieve immune resistance (22). The two most important immune checkpoint pathways are cytotoxic T-lymphocyte-associated antigen 4 (CTLA-4, CD152)/B7 and programmed cell death protein 1 (PD-1, CD279)/programmed cell death ligand 1 (PD-L1) (23). At present, most approved inhibitors exert anti-tumor effects by acting on these two pathways (24). However, few immune checkpoint inhibitors acting on TIGIT, LAG-3, VISTA and other pathways have been developed (25). T cells are activated through a dual-signal pathway. Exogenous and endogenous proteins degraded by lysosomes and the ubiquitin-proteasome system, respectively, form peptides, which bind to the major histocompatibility complex (MHC) on the surface of antigen-presenting cells (dendritic cells, macrophages, B cells, etc.) and tumor cells to form peptide-MHC (PMHC) complexes presented on the cell surface, and then bind to the T cell receptor (TCR) of T cells to generate antigen-specific signals, which constitutes the first signal pathway. Co-stimulatory molecules (CM) CD80 (B7- 1) or CD86 (B7-2) are present on the surface of antigen-presenting cells (APCs) or tumor cells, and bind to the co-receptor CD28 of T cells to generate co-stimulatory signals, which is the second signal pathway (26–31). Co-inhibitory checkpoint molecules CTLA-4 and PD- 1 generate co-inhibitory signals to inhibit the excessive activation of T cells, thereby avoiding the occurrence of autoimmune reactions and damage to normal tissues.CTLA-4 expressed on activated T cells exerts an inhibitory effect at the initial activation stage of T cells by competing with CD28 for B7 proteins, and tumor cells can achieve immune evasion by enhancing the inhibitory effect of CTLA-4.The binding of PD- 1 (expressed on activated T cells, B cells, NK cells and dendritic cells) to PD-L1 (B7-H1, CD274) can down-regulate the activity of signaling pathways such as PI3K/AKT or Ras/MEK/ERK, thereby inhibiting the activation of effector T cells.Tumor cells can overexpress PD-L1 to escape the attack of the immune system (32–43). As shown in **Figure 2**, two signaling pathways involved in T cell activation as well as the two major immune checkpoint pathways, PD- 1/PD- L1 and CTLA- 4/B7, are illustrated.

### 2.2 Tumor microenvironment of gallbladder cancer

**Figure 3:**
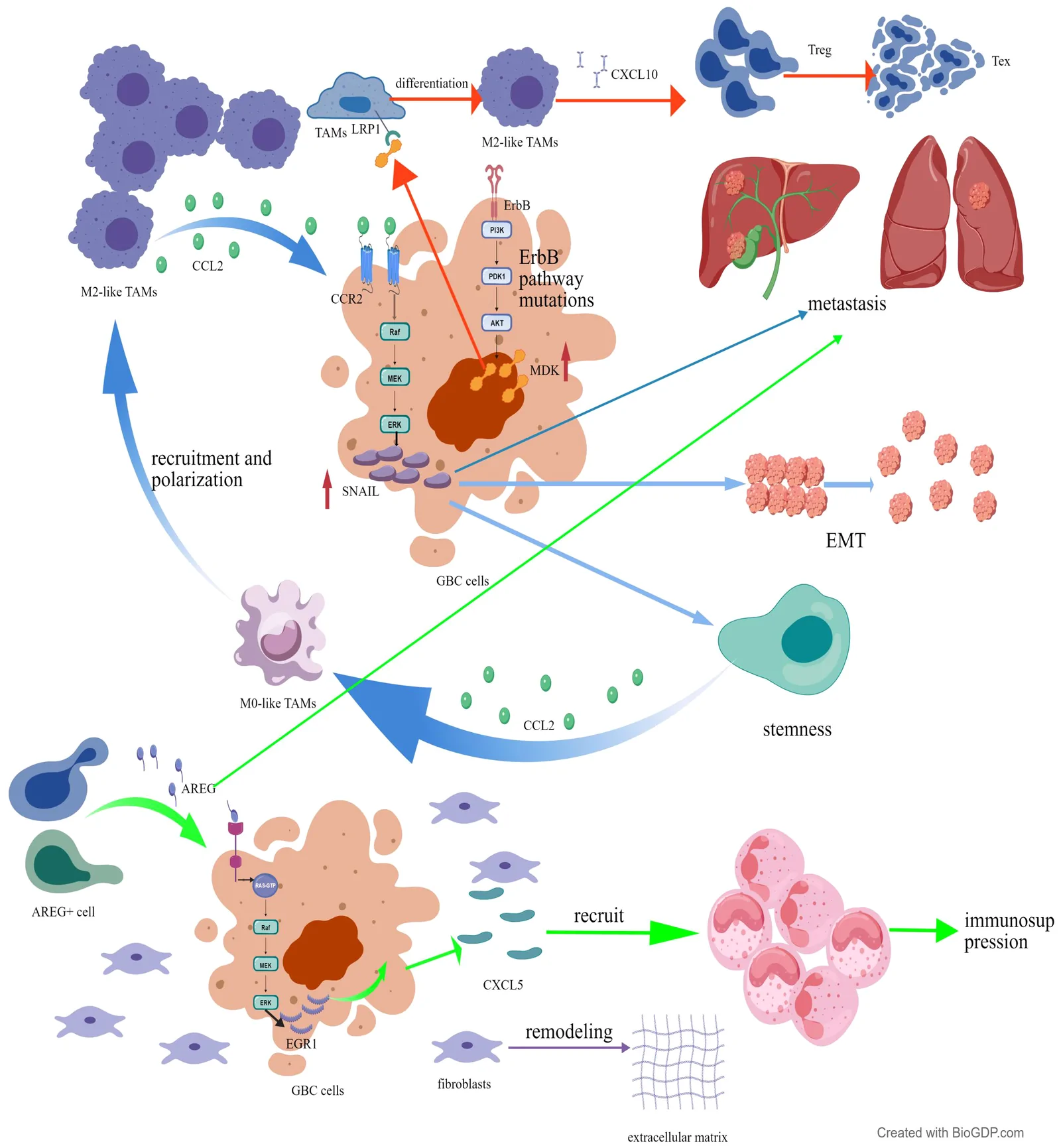
Tumor microenvironment of gallbladder cancer. The blue arrows indicate a vicious cycle where M2-type TAMs secrete CCL2 to activate the MEK/ERK/ELK1/SNAIL pathway in gallbladder cancer cells, facilitating gallbladder cancer metastasis, EMT and stemness, while gallbladder cancer stem cells secrete CCL2 to induce M0-type TAMs polarization and recruit M2-type TAMs. The red arrows show that ErbB pathway mutations trigger M2-type TAMs polarization, which secrete CXCL10 to recruit Treg and Tex. The green arrows demonstrate that AREG+ cells secrete AREG to promote gallbladder cancer metastasis and activate the AREG-CXCL5-neutrophil axis to mediate immune resistance. The purple arrowsrepresent ECM remodeling mediated by CAFs. Created with BioGDP.com.

Patients derive heterogeneous clinical benefits following treatment with immune checkpoint inhibitors (ICIs), which is generally attributed to the influences exerted by the tumor microenvironment (TME) and biomarkers on immune checkpoint pathways.Multiple single-cell and spatial omics studies have identified numerous TME-derived molecules associated with invasion, metastasis and therapeutic resistance in gallbladder cancer. Patients with favorable prognosis of gallbladder cancer exhibit high infiltration of CD8+ T cells, M1 macrophages and NK cells as well as an inflammatory immune phenotype, accompanied by expansion of follicular helper T cells (Tfh cells) and formation of tertiary lymphoid structures (TLSs) after treatment (44–46). Studies have demonstrated that CXCL9+ M1-type tumor-associated macrophages (TAMs) are capable of recruiting CD8+ T cells to the hepatic invasive margin of gallbladder cancer, and patients with high CXCL9 expression achieve a better response to PD- 1 inhibitors (47). Gallbladder cancer harbors an extremely immunosuppressive microenvironment shaped by intricate crosstalk among gallbladder cancer cells, immune cells and the extracellular matrix (ECM). Regulatory T cells (Treg) of the CD4+ lineage, exhausted CD8+ T cells (Tex) (48), M2-type TAMs and other immune populations are abundantly enriched around gallbladder cancer cells, interact with tumor cells to exacerbate the malignant potential of gallbladder cancer, and meanwhile secrete signaling molecules to induce immune resistance in gallbladder cancer. In addition, gallbladder cancer itself possesses multiple targetable pathways that further drive disease progression. The extracellular matrix (ECM) situated between gallbladder cancer cells and immune cells is remodeled into an immunosuppressive state and maintains this suppressive phenotype via reciprocal crosstalk with gallbladder cancer cells. M2-type TAMs secrete chemokine (C-C motif) ligand 2 (CCL2) to activate the MEK/ERK/ELK1/SNAIL signaling pathway in gallbladder cancer cells, thereby promoting malignant phenotypes of gallbladder cancer. Gallbladder cancer stem cells secrete CCL2 to recruit monocyte-derived macrophages and drive their polarization toward M2-type TAMs, establishing a vicious feedback loop (49). Antagonists targeting the CCL2 receptor CCR2 may be combined with ICIs to improve therapeutic efficacy.In addition, gallbladder cancer with ErbB mutations oversecretes MDK to induce polarization of M2-type TAMs.These cells secrete CXCL10 to recruit massive Treg, which further deplete CD8+ T cells and aggravate immunosuppression (50). AREG+ immune cells (T cells, B cells, etc.) also play a pivotal role.Amphiregulin(AREG) secreted by AREG+ immune cells not only facilitates gallbladder cancer metastasis, but also activates the AREG-CXCL5-neutrophil axis to hinder the efficacy of immunotherapy, and blockade of AREG signaling is expected to reverse drug resistance (51). Notably, Targets including OLFM4, ERRα and ASPH in gallbladder cancer upregulate programmed death-ligand 1 (PD-L1) expression to mediate immune evasion, and antagonists developed against these novel targets are expected to enhance the efficacy of immunotherapy (52–54). In addition, cancer-associated fibroblasts (CAFs) endowed with extracellular matrix(ECM) remodeling capacity secrete a variety of stromal components to remodel the immune microenvironment of gallbladder cancer into an immunosuppressive state (55). Furthermore, CAFs interact with gallbladder cancer cells via positive feedback loops including the TSP-4/integrin α2/HSF1/TGF-β axis and mechanotransduction cascades involving tissue stiffness/SEMA7A/integrinβ1/AKT/p300, which collectively drive progressive deterioration of the TME in gallbladder cancer (56, 57). **Figure 3** illustrates the tumor microenvironment of gallbladder cancer.

### 2.3 Biomarkers of gallbladder cancer

**Figure 4:**
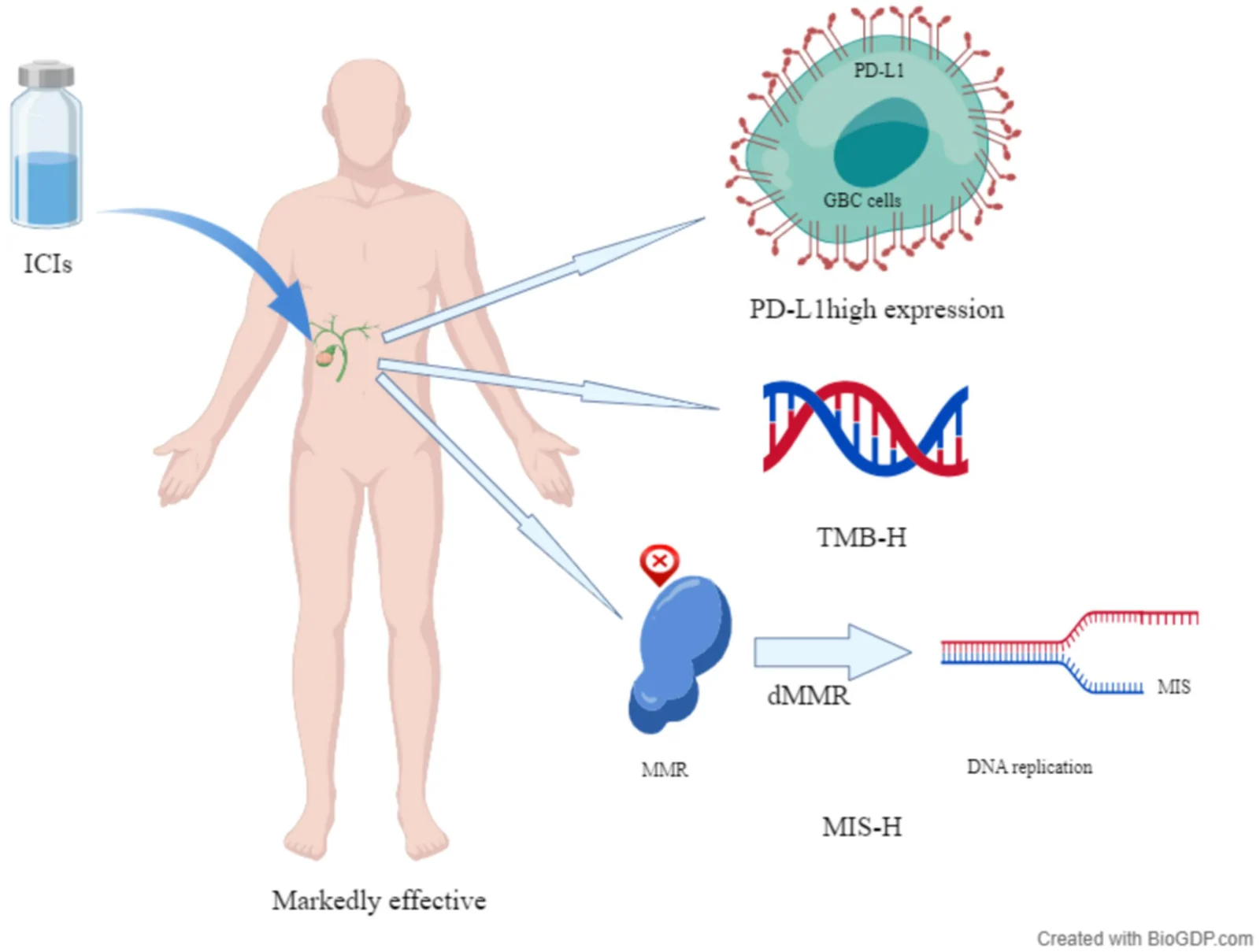
Biomarkers of gallbladder cancer. Patients with gallbladder cancer featuring high PD-L1 expression, TMB-H, and MSI-H/dMMR achieve significant therapeutic efficacy when treated with ICIs. Created with BioGDP.com.

In summary, high PD-L1 expression, high tumor mutational burden (TMB-H), and microsatellite instability-high (MSI-H)/mismatch repair-deficient (dMMR) status are correlated with favorable therapeutic responses (58, 59). PD-L1 acts not only as one of the primary therapeutic targets for gallbladder cancer immunotherapy but also as a predictive biomarker for ICI efficacy. Patients with high PD-L1 expression typically demonstrate improved overall survival (OS), progression-free survival (PFS), and objective response rate (ORR), and those with a high combined positive score (CPS) achieve more favorable prognosis (60–63). TMB is defined as the total number of somatic mutations detected per megabase (1 Mb) of sequenced tumor genomic DNA.Higher TMB levels correlate with superior immunotherapy responsiveness. Based on findings from the KEYNOTE- 158 trial, the U.S. Food and Drug Administration (FDA) approved FoundationOne CDx as a companion diagnostic assay and granted approval to pembrolizumab for the treatment of patients with solid tumors confirmed to harbor TMB-H (≥10 mutations per Mb) via this assay (64–66). Gallbladder cancer frequently harbors somatic mutations including TP53 and ErbB alterations; TP53 mutations correlate with inferior prognosis, whereas gallbladder cancer patients with TMB-H still attain superior clinical endpoints following ICI treatment (67–69). dMMR arises from the inactivation or loss of intracellular mismatch repair proteins, which consequently leads to microsatellite instability (MSI), characterized by length alterations of microsatellite sequences or base mismatches during genomic DNA replication. The MSI-H/dMMR phenotype occurs most frequently in endometrial and colorectal cancers and exhibits a markedly low incidence in biliary tract cancers (BTC), particularly gallbladder cancer.Similarly, gallbladder cancer patients with the MSI-H/dMMR phenotype obtain improved therapeutic benefits from immunotherapy (70–73). **Figure 4** illustrates the Biomarkers of Gallbladder Cancer.

## 3 Clinical trials of PD-1 inhibitors in gallbladder cancer

### 3.1 Pembrolizumab

The global Phase III KEYNOTE-966 trial(NCT04003636) was the world’s first Phase III study of PD- 1 inhibitors for BTC, enrolling a total of 233 patients with gallbladder cancer. No subgroup analyses were performed for endpoints including progression-free survival (PFS), objective response rate (ORR) and adverse events. Only the hazard ratio (HR) for overall survival (OS) in the gallbladder cancer cohort was reported as 0.96 (95%CI: 0.73;1.26). No significant OS benefit was observed in patients with gallbladder cancer compared with those with intrahepatic cholangiocarcinoma (iCCA) (HR = 0.76). Nevertheless, given the significant OS improvement observed in BTC patients as an overall population [HR = 0.83 (95%CI:0.72;0.95; p=0.0034)], we reasonably conclude that pembrolizumab combined with gemcitabine plus cisplatin (GemCis) represents an optional first-line systemic therapy for patients with advanced gallbladder cancer (74). A Phase II clinical trial (NCT03111732) published by Cecilia Monge et al. in 2022 demonstrated that among the gallbladder cancer cohort (n=3) treated with pembrolizumab plus capecitabine and oxaliplatin (CAPOX), 1 patient achieved partial response (PR) and 2 patients had stable disease (SD), with an ORR of 33%. The investigators hypothesized that the enrollment of gallbladder cancer patients might contribute to the favorable overall trial outcomes (75). The LEAP-005 trial (NCT03797326) preliminarily confirmed the promising anti-solid tumor efficacy of lenvatinib combined with pembrolizumab in malignancies including gallbladder cancer, with manageable toxicities (76, 77).

### 3.2 Nivolumab

Klein et al. conducted a subgroup analysis of 13 patients with gallbladder cancer enrolled in the non-randomized Phase II CA209;538 trial (NCT02923934). Under dual immune checkpoint blockade with nivolumab plus ipilimumab, the primary endpoint disease control rate (DCR) was 70% and the ORR was 31%, and no microsatellite-unstable tumors were identified among responding patients (78). The single-arm Phase II basket trial SWOG 1609 (NCT02834013) also evaluated the efficacy of this dual checkpoint blockade regimen in rare malignancies. Its gallbladder cancer cohort recruited 19 pretreated patients with refractory advanced gallbladder cancer, and the trial met its primary endpoint with a confirmed ORR of 16% (79). The latest conference abstract from the MoST-CIRCUIT trial (NCT04969887) reported an ORR of 32% in 23 patients with gallbladder cancer receiving nivolumab plus ipilimumab. Genomic aberration analysis revealed that gallbladder cancer patients were predominantly clustered with features of TP53 mutation and CDKN2A deletion (80). The above studies have demonstrated the favorable efficacy of combined anti-PD- 1/CTLA-4 inhibitor therapy in patients with advanced refractory gallbladder cancer, which warrants further in-depth investigation in future research. Most immunotherapeutic regimens adopt combination strategies. By contrast, a Phase II study (NCT02829918) showed that the ORR was only 15% in gallbladder cancer patients treated with single-agent nivolumab, which was lower than that observed in patients with other subtypes of cholangiocarcinoma (81).

### 3.3 Camrelizumab

The ACCORD randomized controlled trial (NCT04333927) enrolled 93 patients with extrahepatic cholangiocarcinoma (EHCC) and 22 patients with gallbladder cancer who underwent radical surgical resection. Patients in the combination treatment group received camrelizumab plus capecitabine concurrently with radiotherapy. The 3-year overall survival rate was 58.2% in the combination group versus 30.5% in the observation group (HR: 0.43; 95%CI, 0.24;0.79; P = 0.004), and the 3-year recurrence-free survival rate was 40.3% in the combination group versus 17.2% in the observation group (HR: 0.46; 95%CI, 0.28;0.76; P<0.001).This trial demonstrated that camrelizumab combined with chemoradiotherapy improved OS and recurrence-free survival in postoperative patients with EHCC and gallbladder cancer; however, subgroup analyses revealed that patients with gallbladder cancer derived fewer clinical benefits compared with those with EHCC (82). Interim results from an ongoing Phase II trial (NCT05919095) indicated that camrelizumab plus gemcitabine and oxaliplatin (GEMOX) may enable surgical conversion in patients with unresectable gallbladder cancer (83).

### 3.4 Tislelizumab

In the single-arm Phase II ZSAB-TOP trial (NCT05023109), 8 patients with gallbladder cancer received tislelizumab combined with ociperlimab (a TIGIT inhibitor) plus GemCis, achieving an ORR of 100% (95%CI, 63.1;100.0) for the primary endpoint. A total of 75.0% ofthese gallbladder cancer patients were double-positive for TIGIT and PD-L1, and superior therapeutic efficacy was observed relative to patients with iCCA (84). This study illustrates the potential of combined PD- 1/TIGIT inhibitor therapy in the treatment of gallbladder cancer. The prospective Phase II ZSAB-TransGOLP trial (NCT05156788) confirmed that tislelizumab in combination with lenvatinib and GEMOX serves as a feasible conversion therapy regimen for unresectable gallbladder cancer (85).

### 3.5 Sintilimab

A randomized phase II trial conducted by Jingjing Li et al. (NCT04300959) confirmed that sintilimab plus anlotinib combined with GemCis (SAGC) improved PFS and ORR compared with chemotherapy alone (GC). The establishment of orthotopic AKT/YAP-induced cholangiocarcinoma tumor-bearing models demonstrated that incorporating anti-angiogenic agents capable of remodeling the tumor microenvironment into regimens combining PD-1 inhibitors with chemotherapy yielded favorable efficacy, whereas subgroup analyses revealed that the clinical benefit was less prominent in patients with gallbladder cancer relative to those with iCCA (86). Encouragingly, an unpublished single-arm phase II trial (NCT05757336) exclusively enrolls treatment-naive patients with gallbladder cancer to evaluate the efficacy of sintilimab in combination with gemcitabine, nab-paclitaxel and bevacizumab.

### 3.6 Toripalimab

One single-arm phase II study (NCT03796429) investigated the efficacy and safety of toripalimab combined with gemcitabine and tegafur-gimeracil-oteracil potassium (GS). Subgroup analysis demonstrated a median PFS of 7.6 months and a median OS of 15.0 months (95%CI: 11.6;18.4 months) among patients with gallbladder cancer, and worse survival outcomes were observed in patients with PD-L1-negative expression and SMARCA4 mutations (87). Another single-arm phase II study (ChiCTR2100044476) indicated that regimens consisting of PD- 1 inhibitors including toripalimab combined with lenvatinib achieved higher ORR and surgical conversion rates in patients with gallbladder cancer (88). A phase II study involving 4 patients with gallbladder cancer (NCT04217954) reported a median OS of 12.6 months and a median PFS of 8.8 months following treatment with toripalimab plus bevacizumab and hepatic arterial infusion chemotherapy (HAIC), which were shorter than those observed in patients with iCCA and EHCC (89).

## 4 Clinical trials of PD-L1 inhibitors in gallbladder cancer

### 4.1 Durvalumab

TOPAZ- 1 trial (NCT03875235) is the world’s first global phase III study to confirm that first-line immunotherapy combined with chemotherapy significantly prolongs OS of aBTC. A total of 171 cases of gallbladder carcinoma were enrolled, with the durvalumab plus GemCis group (85 gallbladder carcinoma cases) and the placebo plus GemCis group (86 gallbladder carcinoma cases). After initial publication and three years of continuous follow-up, the durvalumab group still improved OS compared with the placebo group, with no new safety events increased, and patients’ quality of life was guaranteed simultaneously (90–93). A number of real-world studies published in recent years have provided reliable clinical evidence (94–98). Notably, the TOPAZ-1 trial released the results of subgroup analysis by primary tumour site. For gallbladder carcinoma, the OS hazard ratio was 0.94 (0.65;1.37), the PFS hazard ratio was 0.90 (0.65;1.24), the ORR was 29.4% in the durvalumab group and 27.9% in the placebo group, and the efficacy was not significant compared with other types ofBTC (99). Overall, durvalumab combined with GemCis is the preferred first-line treatment regimen regardless of the primary tumour site.A small number of trials focusing on immunotherapy combination treatment for gallbladder carcinoma (NCT06214572, NCT06214572, etc.) also deserve our continuous attention.

### 4.2 Atezolizumab

Subgroup analysis of the randomized phase II IMbrave151 trial (NCT04677504) showed that the PFS was 8.5 months in the gallbladder carcinoma group treated with atezolizumab combined with bevacizumab and 6.5 months in the atezolizumab combined with placebo group, with an HR of 0.63 (95%Cl, 0.32~ 1.22), the experimental group can achieve improvement.Meanwhile, it was predicted that the therapeutic benefit in patients with gallbladder carcinoma might be associated with high expression of the VEGFA gene biomarker (100).

## 5 Other PD-L1 inhibitors

A single-arm phase II study (NCT03833661) published by Yoo et al. (2023) demonstrated that for second-line treatment including 32 pretreated patients with gallbladder carcinoma, the ORR of gallbladder carcinoma with the bifunctional fusion protein Bintrafusp alfa was 6.3%. Although the prespecified primary endpoint was not met, the responses were durable to a certain extent and independent of PD-L1 expression, indicating its potential clinical activity (101). Clinical trial data are summarized in **Table 1**.

**Table 1:** Clinical trial.

Immune checkpoint inhibitor	Clinical trial	Dosing regimen	Recruited patients	Primary endpoint	Gallbladder carcinoma subgroup analysis
PD-1 Inhibitor					
Pembrolizumab	NCT04003636 (KEYNOTE-966) (74)	Combined with gemcitabine and cisplatin	BTC:1069 GBC:233	Median OS/months 12.7(Pembrolizumab) 10.9(Placebo)	HR for OS: 0.96 (95%CI: 0.73;1.26) No significant benefit was observed compared with patients with iCCA (HR=0.76)
	NCT03111732 (75)	Combined with capecitabine and oxaliplatin	BTC:11 GBC:3	mPFS:4.1months	ORR:33% GBC patient inclusion associated with good overall outcomes
Nivolumab	NCT02923934 (CA209-538) (78)	Combined with ipilimumab	BTC:39 GBC:13	DCR:44%	DCR:70% ORR:31%
	NCT02834013 (SWOG 1609) (79)	Combined with ipilimumab	GBC:19	ORR:16%	achieve the primary endpoint
	NCT04969887(MoST-CIRCUIT) (80)	Combined with ipilimumab	iCCA:37 GBC:23	ORR:15%	ORR:32% TP53 mutations and CDKN2A deletions were the major genetic aberrations
	NCT02829918 (81)	Monotherapy	BTC:54 GBC:17	ORR:22%	ORR:15%
Camrelizumab	NCT04333927 (82)	Combined with capecitabine and radiotherapy	BTC:93 GBC:22	OS:not reached HR:0.43(95%Cl:0.24-0.79)	Patients with GBC obtained less benefit versus EHC patients
	NCT05919095 (83)	combined with gemcitabine and oxaliplatin	GBC:37	curative resection rate:50%	ORR:50% OS:21.08months mPFS:10.23months
Tislelizumab	NCT05023109(ZSAB-TOP) (84)	Combined with ociperlimab plus gemcitabine and cisplatin	BTC:45 GBC:8	ORR:51.2%	ORR:100% 75.0% of GBC patients were TIGIT and PD-L1 double-positive
	NCT05156788 (ZSAB-TransGOLP) (85)	Combined with lenvatinib plus gemcitabine and oxaliplatin	BTC:41 GBC:3	R0 resection rate:63%	R0 resection rate:100% ORR:67%
Sintilimab	NCT04300959 (86)	Combined with anlotinib plus gemcitabine and cisplatin	BTC:80 GBC:11	mPFS:8.5months(SAGC) 6.3months(GC)	Therapeutic benefit was less pronounced in GBC vs iCCA patients
Toripalimab	NCT03796429 (87)	Combined with gemcitabine and S-1	BTC:50 GBC:20	mPFS:7.0months	mPFS:7.6months Median OS:15.0months
	ChiCTR2100044476 (88)	Combined with lenvatinib	BTC:38 GBC:13	ORR:42.1%	ORR:61.5% surgical conversion rate:46.2% All were superior to other BTC subtypes
	NCT04217954 (89)	Combined with Bevacizumab and HAIC	BTC:32 GBC:4	ORR:84.38%	Median OS:12.6months mPFS:8.8months
PD-L1 Inhibitor					
Durvalumab	NCT03875235 (TOPAZ-1) (90–93)	Combined with gemcitabine and cisplatin	BTC:685 GBC:171	Median OS/months 12.9(Durvalumab)11.3(Placebo)	HR for OS: 0.94 (95%CI: 0.65;1.37) HR for PFS: 0.90 (95%CI: 0.65;1.24) 29.4%(Durvalumab)vs 27.9%(Placebo) Efficacy was not statistically significant compared with other BTC subtypes
Atezolizumab	NCT04677504 (100)	Combined with gemcitabine, cisplatin plus bevacizumab	BTC:162 GBC:43	mPFS/months 8.3(Bevacizumab)7.9(Placebo)	mPFS/months 8.5(Bevacizumab)6.5(Placebo) Benefit may be associated with high VEGFA gene expression
bintrafusp alfa	NCT03833661	Monotherapy	GBC:32	ORR:6.3%	Independent of PD-L1 expression

This table includes partial completed clinical trial data of PD-1 and PD-L1 inhibitors; CTLA-4 inhibitors are not listed independently due to frequent combination with PD-1/PD-L1 inhibitors, and novel immunotherapies are not included due to being in the early research and development stage.

BTC, Biliary tract cancer; GBC, Gallbladder cancer; iCCA, intrahepatic cholangiocarcinoma; EHC, Extrahepatic Cholangiocarcinoma; OS, Overall Survival; mPFS, median Progression-Free Survival; ORR, Objective Response Rate; DCR, Disease Control Rate; HR, Hazard Ratio.

## 6 Other immunotherapies

At present, most studies on gallbladder cancer immunotherapy focus on PD- 1/PD-L1 inhibitors, and other immunotherapies are still in the exploratory stage and have not formed standard treatment regimens. Ipilimumab, a CTLA-4 inhibitor, is the world’s first approved immune checkpoint inhibitor (102, 103), but the efficacy of CTLA-4 inhibitors in gallbladder cancer still needs further research. At present, most studies are conducted in combination with other adjuvant therapies, such as the studies on nivolumab combined with ipilimumab in trials such as CA209;538 mentioned above, or trials of dual immunotherapy with tremelimumab combined with durvalumab plus chemotherapy, ablation and other treatments (104–108). Chimeric antigen receptor T-cell therapy (CAR-T), which has emerged in recent years, has not shown obvious efficacy in solid tumors and is still in the early stage of research and development (109, 110). However, studies have shown that CEA-specific CAR-T cell adoptive transfer therapy is a potential immunotherapy for gallbladder cancer (111), and more studies have adopted a triple combination strategy to prepare super circulating tumor-infiltrating lymphocyte-like cells (ScTILs) to solve the three major obstacles of CAR-T therapy for solid tumors, thus showing efficacy in the treatment of BTC (112).

## 7 Clinical decision-making

### 7.1 Clinical guidelines

It is necessary to note that most internationally recognized authoritative clinical guidelines for gallbladder carcinoma uniformly describe gallbladder carcinoma within BTC, which may be attributed to the fact that most clinical trials enrol BTC as a single study cohort. In particular, to date, only the TOPAZ- 1 trial and the KEYNOTE-966 trial are completed global phase III randomized controlled trials, which is more evident in systemic therapy. Although certain differences in therapeutic efficacy exist across anatomical subtypes of BTC, treatment recommendations with high levels of evidence can still be followed for immunotherapy in patients with gallbladder carcinoma.

The NCCN guidelines recommend durvalumab combined with GemCis or pembrolizumab combined with GemCis as the first-line treatment regimen for unresectable or metastatic BTC with a category 1 recommendation, which is the preferred systemic treatment regimen for aBTC at present. Durvalumab combined with GemCis is also a recommended regimen (category 1) for patients with recurrence more than 6 months after radical surgery and more than 6 months after the end of adjuvant therapy. Pembrolizumab can be used as the first-line or subsequent treatment for patients with unresectable or metastatic MSI-H/dMMR (category 2A) and subsequent treatment for patients with TMB-H (category 2A). Nivolumab combined with ipilimumab is a useful first-line (category 2B) or subsequent treatment for patients with unresectable or metastatic TMB-H under specific circumstances.Dostarlimab-gxly is a category 2B drug for patients with MSI-H/dMMR.Pembrolizumab and nivolumab can be administered via subcutaneous injection.Systemic therapy is recommended for patients with resected gallbladder carcinoma at high risk of postoperative recurrence, and neoadjuvant systemic therapy (Category 2A) is recommended for patients with stage T1b and above or stage T1a with positive resection margins (113–115).

The ESMO guidelines recommend durvalumab combined with GemCis as the first-line treatment regimen for aBTC with evidence level [I, A], a reliable regimen, an ESMO-MCBS V1.1 score of4 and significant clinical benefits. For second-line treatment, pembrolizumab is recommended for MSI-H/dMMR patients with disease progression or intolerance to previous treatment[III, A; ESMO-MCBSv1.1score: 3] (116, 117). In addition, the European Medicines Agency (EMA) approved pembrolizumab combined with GemCis as a new first-line treatment regimen [I, A] (118, 119). The Pan-Asian adapted ESMO Clinical Practice Guidelines for the management of BTC in Asian patients reached a consensus on the above regimens (120).

**Figure 5:**
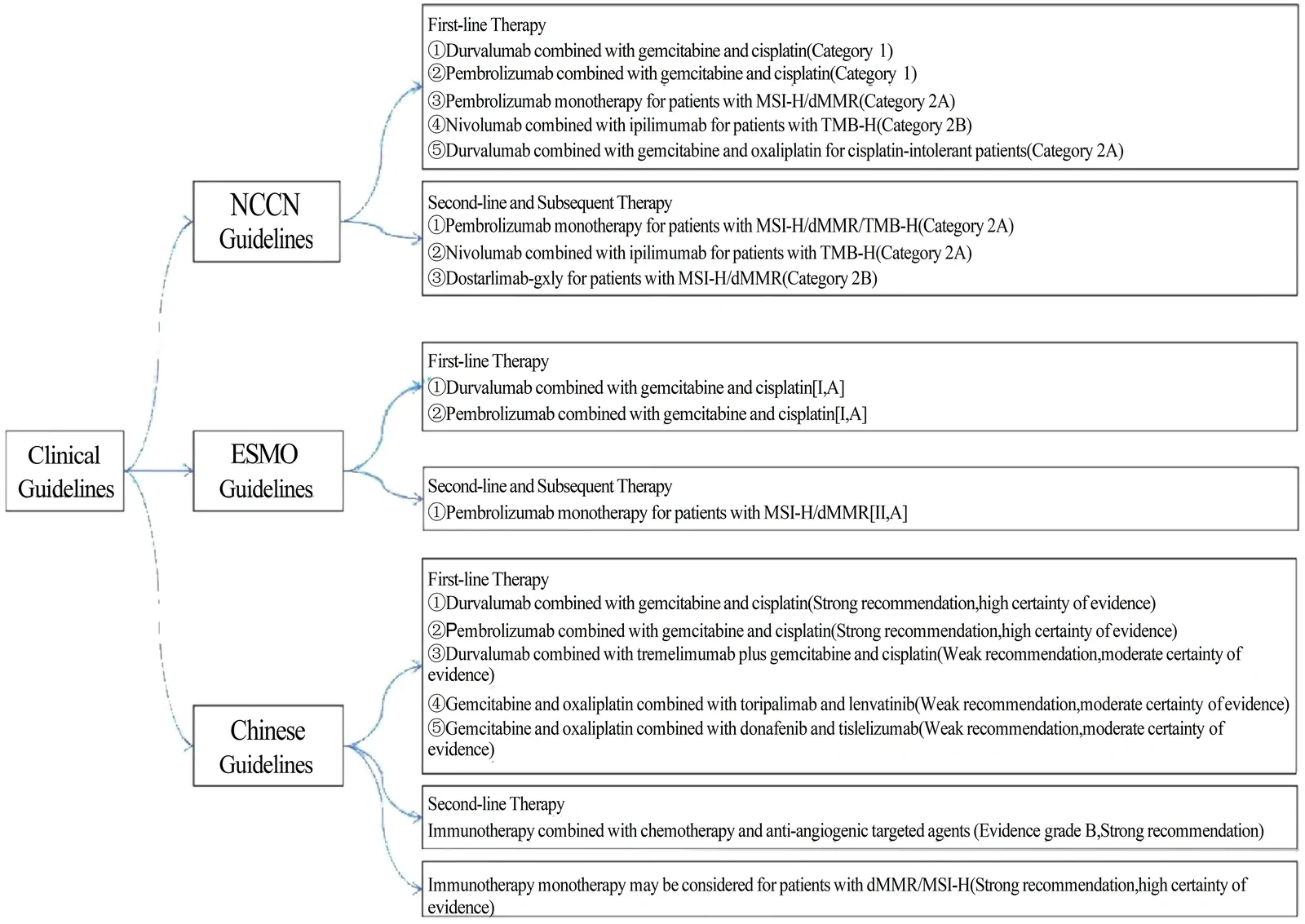
Clinical guidelines. Source: The author.

Several major Chinese guidelines strongly recommend immune checkpoint inhibitor combined with chemotherapy regimens, and immunotherapy combined with chemotherapy and anti-angiogenic targeted drugs is an optional treatment. Among them, durvalumab combined with GemCis and pembrolizumab combined with GemCis are the first-line systemic treatment regimens for BTC patients with good performance status (PS 0- 1) (strong recommendation, high certainty of evidence).GemCis combined with durvalumab and tremelimumab, GEMOX combined with toripalimab and lenvatinib, GEMOX combined with donafenib and tislelizumab are all considerational regimens (weak recommendation, moderate certainty of evidence).For dMMR/MSI-H patients, immunotherapy monotherapy may be considered (strong recommendation, high certainty of evidence) (121–123). As shown in **Figure 5**, the recommended regimens of immune checkpoint inhibitors in systemic therapy mentioned in clinical guidelines from the United States, Europe, and China are presented.

Other guidelines are formulated according to the clinical trial conditions, accessibility of immune drugs, expression of tumor markers and other factors in their respective countries, but all support durvalumab combined with GemCis and pembrolizumab combined with GemCis as the first-line treatment regimens for aBTC patients (124–126).

### 7.2 Approval status

Currently, the FDA has only approved pembrolizumab in combination with GemCis for the treatment of adult patients with locally advanced unresectable or metastatic BTC, as well as adult and pediatric patients with pretreated refractory solid tumors without alternative treatment options who have TMB-H or MSI-H/dMMR status (127). Durvalumab in combination with GemCis is approved for adult patients with unresectable or metastatic BTC (128). The NMPA has approved pembrolizumab combined with GemCis for first-line treatment of patients with locally advanced or metastatic BTC, and single-agent pembrolizumab for adult patients with unresectable or metastatic MSI-H/dMMR pan-solid tumors (129). Durvalumab in combination with GemCis is approved for first-line treatment of adult patients with locally advanced or metastatic BTC (130).

### 7.3 Clinical experience

Most patients with gallbladder cancer are asymptomatic and present to the Department of Hepatobiliary Surgery due to the detection of space-occupying lesions in the gallbladder during examinations. This group of people generally has abdominal ultrasound results, and further examinations such as contrast-enhanced abdominal magnetic resonance imaging, contrast-enhanced abdominal computed tomography, and tumor markers should be completed to initially assess the benignity or malignancy of the gallbladder space-occupying lesion, the presence or absence of metastases to organs such as the liver and lungs, the presence or absence of lymph node metastasis, and the presence or absence of portal vein system invasion and tumor thrombus formation. Patients with clear surgical indications undergo surgery according to the stage; the surgical resection specimens are sent to the Department of Pathology for histopathological examination to confirm the pathological type, degree of differentiation, stage, and immunohistochemistry. To guide systemic treatment, routine detection items should include PD-L1 expression, dMMR, mutation targets, tumor-infiltrating cells, etc. If conditions permit, further detection of MSI and TMB should be performed. Patients after radical resection of gallbladder cancer are not within the scope of indications for the two currently approved ICIs, and patients can be recruited into clinical trials to receive ICI treatment with their consent. For patients who are not eligible for surgical resection, puncture biopsy of the gallbladder and metastatic sites should be performed. Based on the comprehensive pathological examination results, imaging and laboratory test results, the patient’s physical condition and economic status, and medical insurance policies, pembrolizumab or durvalumab combined with GemCis systemic treatment is preferred for the patient.

Symptomatic patients with gallbladder cancer often have advanced disease and seek medical attention with complaints such as abdominal pain, jaundice, fever, and poor appetite. The diagnosis and treatment process is similar to that of asymptomatic patients, but patients with severe symptoms cannot tolerate ICI treatment and need to first receive symptomatic treatment such as biliary puncture drainage combined with liver-protective drugs to improve liver function and reduce jaundice, antibiotics to control infection, and parenteral nutrition or enteral nutrition to improve nutritional status.

**Figure 6:**
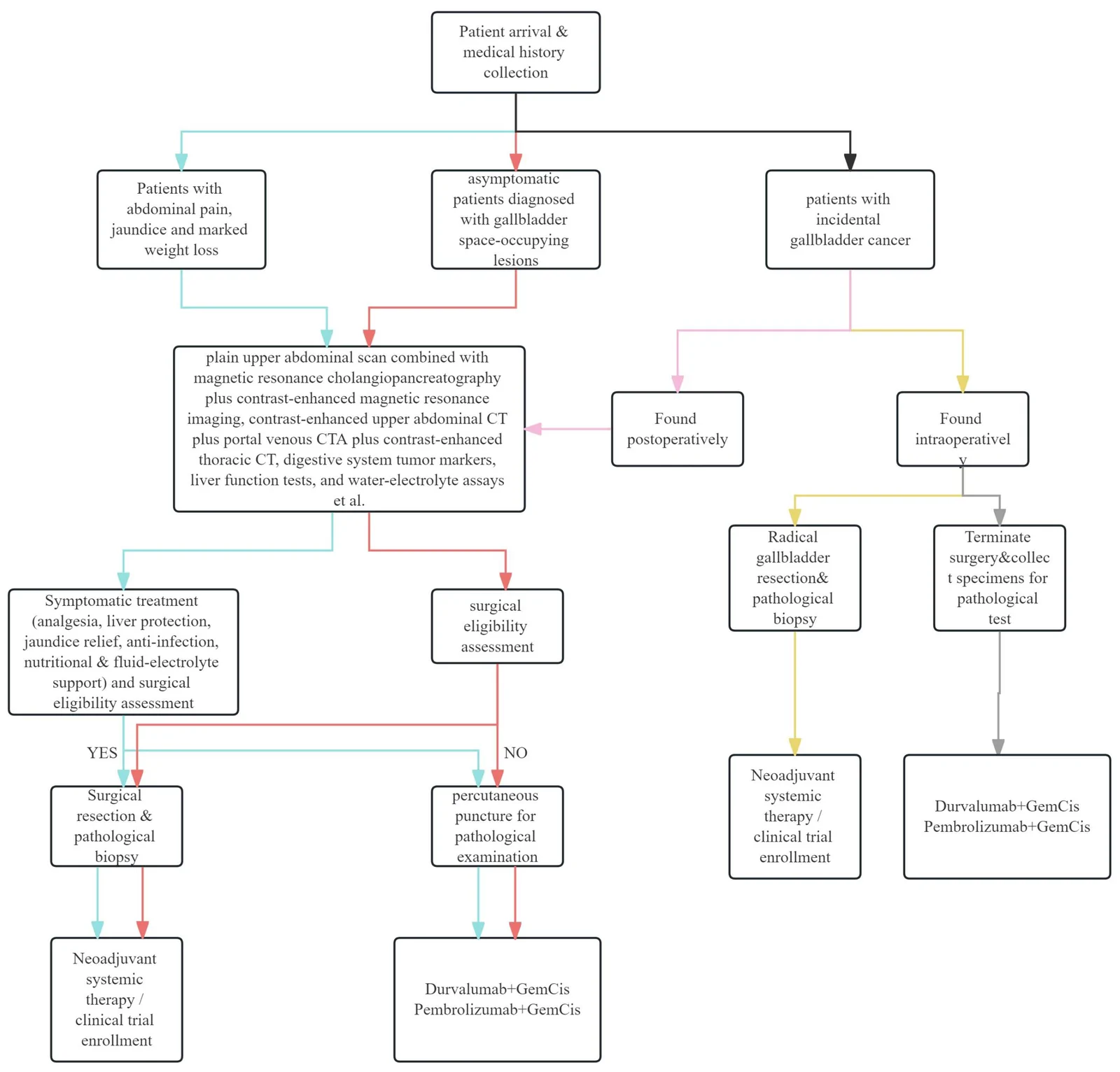
Clinical immunotherapy flow chart. Source: The author.

Incidentally discovered gallbladder cancer is mostly detected during cholecystectomy performed for benign diseases such as gallstones and gallbladder polyps. Patients diagnosed with gallbladder cancer by postoperative pathological examination need to be readmitted for reoperation, postoperative neoadjuvant systemic therapy, or receive ICI therapy in accordance with unresectable or metastatic gallbladder cancer. Patients diagnosed with gallbladder cancer by intraoperative frozen section can mostly be converted to radical cholecystectomy during the operation, and ICI therapy is administered postoperatively as appropriate. As shown in **Figure 6**, we illustrated the clinical diagnosis and treatment workflow of immunotherapy for gallbladder carcinoma.

## 8 Discussion

Since CTLA-4 and PD-1 were discovered and their mechanisms elucidated respectively in the 1890s, ICIs have gradually evolved into the most successful and widely adopted therapeutic strategy for cancer immunotherapy. Ipilimumab, a CTLA-4 inhibitor which initiated the world’s first clinical trial in 2000, became the first globally approved immune checkpoint inhibitor in 2011, followed by extensive approvals of PD-1 inhibitors and PD-L1 inhibitors. Over more than three decades of development, research on ICIs has advanced to the development of novel agents targeting next-generation immune checkpoints including TIGIT, TIM3 and LAG3, while researchers are also exploring next-generation precision immunotherapeutic drugs targeting TME, biomarkers and CAR-T therapies (131–134). With continuous advances in detection technologies such as immunohistochemistry, multiplex immunofluorescence, next-generation sequencing (NGS), PCR fragment analysis and liquid biopsy (135–137), an increasing number of immunotherapy clinical trials in recent years have incorporated assessments of biomarkers and the tumor microenvironment, aiming to clarify the mechanisms by which PD-L1 expression, MSI-H/dMMR phenotype, TMB, tumor-infiltrating lymphocytes, TAMs, Treg, Tex and CAFs influence the efficacy of ICIs, and to identify novel therapeutic targets for developing new drugs to improve patient prognosis. This indicates that neoadjuvant systemic therapy is evolving toward personalization, precision and high efficiency.

As a rare type of solid malignant tumor of the digestive system, BTC has been investigated in numerous clinical trials in recent years with encouraging outcomes, providing experimental evidence for the approval of ICIs for BTC and updates of clinical guidelines. Nevertheless, research evidence for BTC remains insufficient compared with extensively studied malignancies with robust clinical data such as non-small cell lung cancer, melanoma and triple-negative breast cancer (138–140). Current BTC studies are predominantly phase I and single-arm phase II trials, a considerable number of which are still ongoing, with only the TOPAZ- 1 trial and KEYNOTE-966 trial serving as global phase III placebo-controlled trials. As one anatomical subtype of BTC, gallbladder carcinoma suffers from a scarcity of clinical trials and relatively low research attention. Notably, multiple trials have performed subgroup analyses stratified by anatomical subtype to report immunotherapy efficacy data for gallbladder carcinoma and compare therapeutic outcomes across different BTC subtypes, among which the TOPAZ- 1 trial is included. We observed that patients with gallbladder carcinoma derive less clinical benefit from ICIs relative to other BTC subtypes, particularly iCCA. This phenomenon may be attributed to the markedly immunosuppressive and pro-metastatic TME in gallbladder carcinoma, which is shaped collectively by abundant infiltration of M2-type TAMs, CAFs with extracellular matrix (ECM) remodeling capacity, enriched Treg and Tex cells, AREG+ immune cells capable of recruiting neutrophils to mediate immune resistance, and intricate intercellular crosstalk, as well as the biomarker landscape featuring low prevalence of MSI-H/dMMR phenotypes and high frequency of poor-prognosis mutations such as TP53. However, clinical studies evaluating nivolumab combined with ipilimumab and tislelizumab combined with ociperlimab have demonstrated better efficacy in gallbladder carcinoma, which may be explained by the concurrent expression of multiple therapeutic targets in this tumor type. Additional clinical trials investigating dual immunotherapy regimens are warranted to further validate therapeutic efficacy in the future. We also noted that combination therapy with ICIs yields favorable efficacy in the conversion therapy for patients with gallbladder cancer. Obtaining surgical access generally indicates a higher survival rate, hence this represents a research direction worthy of investigation.

Currently, only pembrolizumab and durvalumab have obtained global approval for advanced unresectable or metastatic BTC, leaving clinicians with limited therapeutic options. The modest efficacy observed for gallbladder carcinoma in subgroup analyses also creates clinical dilemmas for both physicians and patients. Accordingly, pathological confirmation and biomarker testing should be routinely implemented in clinical immunotherapy for gallbladder carcinoma, and the optimal systemic regimen dominated by immunotherapy combined with chemotherapy should be selected based on individual clinical characteristics. For patients with gallbladder carcinoma who do not meet the approved indications for pembrolizumab and durvalumab, alternative therapeutic modalities including chemoradiotherapy and targeted therapy can be considered, and enrollment in ongoing clinical trials is also a recommended option.

## 9 Conclusion

Patients with gallbladder carcinoma can benefit from immune checkpoint inhibitor therapy. Given the current situation that various immunotherapy combination regimens have achieved therapeutic efficacy and the underlying mechanisms are continuously being elucidated, it is time to conduct further research on patients with gallbladder carcinoma as an independent cohort to generate higher-level evidence for immunotherapy of gallbladder carcinoma.
